# Role of socioeconomic deprivation on inequities in care outcomes of haematological malignancies treated with stem cell transplant in the UK: a systematic review protocol

**DOI:** 10.1136/bmjopen-2025-111282

**Published:** 2026-07-10

**Authors:** Zareen Deplano, Samuel Cusworth, Carolyn Doree, James Griffin, Nicola Adderley, Joht Singh Chandan

**Affiliations:** 1 Department of Applied Health Sciences, University of Birmingham, Birmingham, UK; 2 Cell, Apheresis & Gene Therapies, NHS Blood and Transplant, Bristol, UK; 3 Blood and Transplant Research Unit (BTRU) in Precision Transplant and Cellular Therapeutics, University of Birmingham, NIHR, Birmingham, UK; 4 Systematic Review Initiative, NHS Blood and Transplant, Bristol, UK; 5 Birmingham Biomedical Research Centre, National Institute for Health and Care Research, Birmingham, UK

**Keywords:** HAEMATOLOGY, Bone marrow transplantation, Systematic Review, Health Equity, Neoplasms, Protocols & guidelines

## Abstract

**Abstract:**

**Introduction:**

Stem cell transplant (SCT) is a potential cure for haematological malignancies whose outcomes are predicted to be poor on chemotherapy alone and cases difficult to treat using standard of care treatment. There is growing evidence to show that not all patients who would benefit from SCT receive it due to inequities in the treatment pathway. The role of socioeconomic deprivation on inequities in the SCT setting is unclear due to conflicting findings. We aim to synthesise the UK evidence base in this area from the point of diagnosis to transplant to guide further research and policy to improve survival for underserved patients.

**Method and analysis:**

Primary electronic searches will be performed in bibliographic databases including MEDLINE, Embase, Proquest Databases, Central & CDSR, Stem Cell Evidence and Web of Science and Google Scholar for grey literature. Eligibility criteria will include observational studies of UK patients diagnosed with haematological malignancies treated with SCT, where outcomes are reported according to socioeconomic deprivation. Two independent reviewers will screen each study and assess the quality of all included studies using the Newcastle-Ottawa grading system. Outcomes of interest include delay in diagnosis/referral, treatments/interventions received, referral to transplant, presence of comorbidities affecting transplant eligibility, transplant utilisation rates, waiting time to transplant and survival rates. Post-transplant outcomes are beyond the scope of this review. Inequities in SCT due to socioeconomic deprivation will be narratively summarised. Meta-analysis will be conducted dependent on data availability. This protocol has been registered with PROSPERO (2024: CRD42024620592).

**Ethics and dissemination:**

Ethical approval is not required for this study as it is a secondary analysis of published data.

We are not aware of any ethical concerns regarding SCT or treatment of donors in the studies included in this research. This systematic review will retrieve, screen and extract data from UK research. SCT and the treatment of donors in the UK follow the National Health Service (NHS) and NHS Blood and Transplant guidelines and policies and are regulated by the Human Tissue Authority and Medicines and Healthcare Products Regulatory Agency. The data which will be used in this review will consist of data from NHS digital/NHS England or single NHS centre data, which will follow the guidelines and regulations above.

The findings will be submitted for peer-reviewed publication, shared at conference presentations, and disseminated to both clinical and patient groups including National (Black, Asian, Mixed Race and Minority Ethnic) BAME Transplant Alliance and the National Institute for Health Research Blood and Transplant Research Unit in Precision Transplant and Cellular Therapeutics.

**PROSPERO registration number:**

CRD42024620592.

**STRENGTHS AND LIMITATIONS OF THIS STUDY**

- This review has a detailed and comprehensive search strategy, using a variety of search tools and wide range of databases.
- The Ovid Limits filter ‘Remove MEDLINE Records’ will be applied to the Embase search in order to reduce the retrieval of irrelevant results and maintain precision; it is possible that a small number of relevant records may be excluded as a result.
- This review will focus on data collected from studies performed in a UK population only. The findings can be applied to a UK population but application to other countries will be limited.
- Patients and the public participated in the development of the review and will support interpretation of findings and dissemination to the wider public.
- Due to resource constraints, non-English language studies will be excluded.

## Introduction

 Haematological malignancies are cancers of the blood (leukaemia), lymph nodes (lymphomas—Hodgkin and non-Hodgkin) or bone (myelomas).^1 2^ They represent the fifth most common cancer type and the third largest cause of cancer-related death, with 41 000 diagnoses and 16 000 deaths yearly in the UK.^3^,^5^ Stem cell transplantation (SCT) is a potentially curative treatment for those patients with poor prognosis on chemotherapy alone and cases difficult to treat using standard of care treatment.^6^,^8^ The most common clinical indications for SCT are acute myeloid leukaemia (AML), acute lymphoid leukaemia (ALL), myelodysplastic syndrome (MDS) and myeloma.^9^ In adults with AML in first complete remission, SCT has been shown to reduce the risk of disease relapse by 60% compared with intensive chemotherapy alone.^10^ A systematic review and meta-analysis by Koreth *et al*, analysing 24 trials and 6007 patients, found that SCT led to significant relapse-free survival and overall survival (OS) benefits for AML patients with unfavourable genetic mutations and high chance of relapse.^8^

Health inequities defined as unfair and avoidable differences in health between social groups are evidenced by the 8–10 year gap in life expectancy and 20-year gap in healthy life expectancy between the most and least deprived areas in the UK.^11^ Patients living with cancer in deprived areas are 60% more likely to die than those from more affluent areas.^12^ In the haematological malignancies setting, inequities have been reported to impact on incidence and prevalence of disease, diagnosis, referral and treatment outcomes. Age-standardised incidence rates of myeloma have been reported to be 2.7–3.0 times higher in people of Black ethnicity compared with white ethnic groups in England.^13^ Despite this, ethnic minority patients are reported to experience delayed diagnosis and referral.^14^,^16^ A survey by the Blood Cancer Alliance showed that 45.5% of ethnic minority patients visited their general practitioner (GP) three or more times before being referred compared with 25% of blood cancer patients overall.^14 17^ An increased incidence of childhood leukaemia in more affluent communities suggests under-diagnosis in the poorest as one of the possible explanations.^18 19^ Socioeconomic status has been reported to strongly influence outcomes of haematological malignancies. Fegan *et al* showed significantly better survival in the least deprived group, with a 41% lower risk of mortality compared with the most deprived group of patients with chronic lymphocytic leukaemia.^20^ Similarly, Bhayat *et al* reported poorer survival in AML, with 50% higher mortality rates in patients from the most deprived quintile compared with the least deprived.^21^

Despite the survival benefits of SCT, there is growing evidence to suggest that not all patients who could benefit from SCT receive it, with inequities experienced along the SCT treatment pathway, from diagnosis, transplant referral and eligibility assessment to donor identification and transplant.^15 22^ An inquiry from the All-Party Parliamentary Group on SCT access described geographical, cultural, complex health systems, housing, health literacy and education, race, ethnicity, discrimination and socioeconomic deprivation as barriers faced by patients accessing SCT treatment and care.^15^

Socioeconomic deprivation refers to a lack of income and other resources needed to fully participate in society. Measures of socioeconomic deprivation can be collected at the individual, household (income, education and occupation) or area level using metrics such as crime, housing, access to local services and living environment.^23 24^ While socioeconomic deprivation is a key driver of health inequities and a fundamental social determinant of health, modifiable by policy and directly tied to the National Health Service (NHS’s) Long-term plan and Core 20plus5 agenda, studies on its role in SCT access and survival have yielded mixed results.^25^,^30^ In their 2024 study of AML patients who underwent SCT, Oliveri *et al* showed limited impact of socioeconomic and other non-biologic characteristics such as race/ethnicity and distance travelled on non-relapse mortality (NRM), relapse-free survival and OS in multivariate analysis except for marital status with improved NRM (risk of NRM HR=0.70 (95% CI 0.50 to 0.97, p=0.031) and having no insurance with greater risk of death (HR=1.49 (95% CI 1.05 to 2.12, p=0.024)).^1^. In contrast, studies exploring SCT access suggest that poverty, lack of insurance and education have an impact on transplant rates with lower transplant rates described with higher poverty.^31^,^33^ The evidence in this area is mostly US based, where the insurance-based health system represents an important barrier to healthcare, contributing to health inequities.

In 2022, the UK Stem Cell Strategic Forum published a 10 year report for SCT, recommending the need for better understanding of the role of ethnicity and socioeconomic factors on patient access to SCT and outcomes.^34^ Ethnicity is also a major determinant of health inequity and a well-known barrier to finding an optimal donor source for SCT, especially for patients from an ethnic minority background.^35^,^37^ Socioeconomic deprivation and ethnicity are linked and intersect.^38^ In 2019, people from all ethnic minority groups (except Indian, Chinese and White Irish) were more likely than White British people to live in the most overall deprived 10% of neighbourhoods in England.^39^ Ethnicity remains an important area of investigation and while our recent systematic review protocol highlighted the gaps in the UK evidence base of ethnic inequities in SCT, this review will focus on the role of socioeconomic deprivation.^40^

### Why is it important to do this review?

Addressing health inequities is a priority for health services in the UK and beyond. To achieve this, the factors influencing health inequities in different clinical settings and regions need to be reported and their role/impact on outcomes described, to help interventions to be put in place to level up, assess and guide future research.

There are conflicting results in the literature on the impact of socioeconomic deprivation on SCT access and survival in the haematological malignancy setting. Most of the data in this setting has been conducted in the USA, where the insurance-based healthcare system is a significant barrier to healthcare access, limiting applicability of findings to other healthcare systems. To our knowledge, no systematic review has been undertaken to synthesise the evidence of the role of socioeconomic deprivation on inequities in the SCT treatment pathway, where healthcare is free from the point of need, such as the UK. This review will focus on studies using UK data only due to differences in societal make-up, healthcare infrastructure and drivers of deprivation, which vary from country to country. Identification of the socioeconomic disparities in this UK setting is crucial to help identify inequity in care provision and set policy to address those inequities and improve survival for underserved patients.

### Aims

This systematic review aims to describe the inequities due to socioeconomic deprivation at all stages of haematological cancer care for those haematological malignancies treated by SCT, in the UK.

### Objectives of this systematic review

1. Investigate the role of socioeconomic deprivation at different stages of the treatment pathway from the point of diagnosis to treatments/interventions received, referral to transplant, transplant eligibility, transplant utilisation rates, waiting time to transplant and survival rates. Post-transplant outcomes are beyond the scope of this review.
2. To describe patterns and evidence gaps that may indicate potential mechanisms underlying socioeconomic inequities across the SCT pathway.
3. To describe the association between inequities in the SCT treatment pathway and socioeconomic deprivation.

## Methods and analysis

Standard systematic review methodology will be used in accordance with the Preferred Reporting Items for Systematic Reviews and Meta-Analyses (PRISMA) guidelines and in line with the Cochrane Handbook.^41 42^ This protocol is registered with PROSPERO (2024: CRD42024620592) and has been written following PRISMA Protocols and checklist (online supplemental file 1).^43^

We plan to start the systematic review in September 2025 and finish in June 2026.

### Eligibility criteria

This review is intentionally focused on quantitative observational evidence, including cohort, cross-sectional, case-crossover and case–control studies, to characterise the distribution and magnitude of inequities across the SCT pathway within the UK. Qualitative and mixed-method research will be excluded due to resource constraints, although we acknowledge that exclusion of qualitative and mixed-methods studies limits the ability to make causal inferences about mechanisms underlying socioeconomic inequities. Systematic reviews and research syntheses will be included and used to identify primary studies, as a source of references. They may also be used in the introduction and/or discussion of the review.

As this review is focused on inequities in access to SCT from the point of diagnosis to transplant, post-transplant outcomes will be out of scope and excluded. We will include studies which include a UK population, with non-UK populations excluded due to differences in health systems, drivers of socioeconomic deprivation, education, cultures, behaviours and economic conditions of different nations.

In this review, socioeconomic deprivation will be defined using validated measures and grouped a priori into: (1) area-level composite indices (eg, Index of Multiple Deprivation (IMD) and national equivalents) (2) area-level material deprivation indices (eg, Townsend, Carstairs) and (3) individual-level socioeconomic indicators (eg, income, education, occupation).^44^

All inclusion and exclusion criteria are detailed in table 1.

**Table 1:** Inclusion and exclusion criteria

	Inclusion	Exclusion
Population	UK patients with haematological cancers listed as clinical indications for SCT (BSBMTCT).	Non-UK population Non-haematological (blood) malignancies Non-malignant haematological conditions (immunodeficiency, blood disorders, eg, sickle cell disease, beta-thalassaemia), bone marrow failure (aplastic anaemia)
Exposure	High socioeconomic deprivation, low socioeconomic status (SES)	
Comparator	Low deprivation, high SES	
Outcomes	Reports on one of the outcomes of interest: delay in diagnosis/referral, treatments/interventions received, referral to transplant, presence of co-morbidities affecting transplant eligibility, transplant utilisation rates, waiting time to transplant, survival/mortality rates	Post-transplant (Graft versus host disease and infection) Chimeric antigen receptor T cell therapy
Research type	Observational study design, including cross-sectional studies, case–control studies, case-crossover, cohort studies; systematic reviews and research syntheses	Qualitative studies Animal studies Cell/molecular studies/testing Drug efficacy and safety studies Incidence/risk of cancer Morbidity/prevalence
Language	English only	Non-English language studies

BSBMTCT, British Society of Blood and Marrow Transplantation and Cellular Therapy; SCT, stem cell transplant.

UK patients with haematological cancers which are clinical indications for SCT as listed by the British Society of Blood and Marrow Transplantation and Cellular Therapy (BSBMTCT) will be included.^45^ This classification of haematological cancers was used due to the focus of this review on a UK population. These haematological malignancies are:

- Leukaemia
  - Chronic myeloid leukaemia
  - AML
  - ALL
- Lymphomas
  - Hodgkin’s lymphoma
    - Classical Hodgkin’s lymphoma
    - Nodular lymphocyte-predominant B-cell lymphoma
  - Non-Hodgkin’s lymphomas
    - Peripheral T cell lymphoma
    - Diffuse large B-cell lymphoma
    - Mantle cell lymphoma
    - Follicular lymphoma
    - Burkitt lymphoma
    - Marginal zone lymphoma
    - Primary central nervous system lymphoma
    - Extranodal marginal zone lymphoma of mucosa-associated lymphoid tissue lymphoma
    - Acute lymphoblastic lymphoma
    - Lymphoplasmacytic lymphoma
    - Waldenström’s macroglobulinaemia
    - Chronic lymphocytic leukaemia/small lymphocytic lymphoma (Richter’s syndrome).
- Plasma cell dyscrasias
  - Myeloma (multiple myeloma)
  - Amyloid light chain (AL) (AL amyloidosis)
  - Polyneuropathy, organomegaly, endocrinopathy, monoclonal gammopathy and skin changes syndrome
- Myelofibrosis (primary/secondary)
- MDS (myelodysplasia, myelodysplastic neoplasm).

### Information sources

The following databases and search tools will be searched from inception to August 2024 for relevant evidence: MEDLINE (Ovid, 1946 onwards), Embase (Ovid, 1974 onwards), Cochrane Central Register of Controlled Trials (CENTRAL) and Cochrane Database of Systematic Reviews (CDSR) (The Cochrane Library, Wiley), APA PsycInfo (Proquest, 1806 onwards), Public Health, Psychology and Nursing & Allied Health Databases (Proquest, 1946 onwards), British Nursing Index (Proquest, 1994 onwards), Publicly Available Content Database (Proquest), Stem Cell Evidence (Evidentia, 1980 onwards), Web of Science: All Databases except MEDLINE (Clarivate, 1900 onwards), ClinicalTrials.gov and WHO International Clinical Trials Registry Platform (ICTRP) (online supplemental file 2).

Additionally, we will search Google Scholar (GS) using the advanced search feature and the search string detailed below in the ‘Search Strategy’ section to identify grey literature listed in English (first 10 pages).

Methodology papers recommend screening the first 200–300 GS results.^46 47^ Our lower threshold (first 100 results) was selected based on our available resources and the use of other grey literature sources, namely ProQuest Dissertations & Theses Citation Index.

We will also manually check references lists and citations of eligible studies to capture additional data to ensure full coverage of eligible literature.

### Search strategy

A search strategy (online supplemental file 2) has been developed by co-author CD broadly including the terms “Socioeconomic Factors”, “health Inequities”, “health Services Accessibility”, “Social Determinants of Health”, “Social Conditions”, “Social Socioeconomic deprivation”, “Social Environment”, “Sociodemographic Factors”, “Social Isolation”, “Social Marginalization”, “Social Vulnerability”, “Stem Cell Transplantation”, Bone Marrow Transplantation”, “Cell Transplantation”, “Hematologic Neoplasms”, “Lymphoma, Leukemia”, “Neoplasms”, “Plasma Cell”, “Bone Marrow Diseases”, “Great Britain” and “United Kingdom”.

Studies in languages other than English will be excluded due to resource constraints.

Google Scholar search string: ((deprivation OR socioeconomic) OR (determinants AROUND (1) health) OR (inequities OR inequalities OR access)) AND ((((haematological OR blood) AROUND (1) (cancer OR malignancies)) OR lymphoma OR leukaemia OR myeloma OR myelodysplastic) OR ((“bone marrow transplant” OR “stem cell transplant” OR “haematopoietic cell transplant”) OR (BMT OR SCT OR HSCT))) AND (“united kingdom” OR england OR uk OR britain).

### Study records and data management

Covidence will be used to conduct and organise reviewing of the searched evidence, including removal of duplicates.^48^ Mendeley Reference Manager will be used to organise the literature extracted after full-text review.^49^ This will also be used to automate the downloading of open-access papers. The PRISMA flow diagram will be used to summarise the screening and selection of the studies.^50^

### Study selection process

Two independent reviewers will conduct title and abstract screening according to the predefined inclusion/exclusion criteria (table 1). The outcomes (eg, delayed diagnosis, referral patterns, comorbidities) upstream of transplant eligibility may not be captured in SCT-focused datasets and are more likely to be informed by distinct and non-overlapping study populations. Our screening has been designed to include studies reporting on haematological malignancies and/or SCT study populations to ensure we include all available data. Any disagreement unresolved through discussion will be evaluated by a third party (JSC or NA). Full texts of the shortlisted articles will be obtained and exclusion criteria applied by two reviewers (ZD, SC) based on the above inclusion and exclusion criteria, with an additional reviewer providing support to resolve any disagreements (JSC or NA). The final list of eligible studies will be obtained for inclusion in the review after the full text review.

Following Cochrane handbook guidance, we will compare multiple publications from the same study based on criteria including the authors’ names, location/setting, number of participants, baseline data and date and duration of the study. If the publications are identified to be from the same study, they will be merged into one study record, data will be extracted from both, we will note any inconsistencies, and the authors will be contacted.^51^

### Data collection

The identified studies meeting the eligibility criteria will be inputted into the data extraction form, which uses a modified Cochrane Public Health Group Data Extraction and Assessment Template. The data extraction form will be piloted by two reviewers to provide an opportunity to refine the data extraction process and ensure the relevant data items are being collected (online supplemental file 3). Data extraction will be done independently, in duplicate, by ZD and SC.

Any discrepancies in data collection will be resolved through discussion between the reviewers and the project leads (JSC and NA).

### Data items

For each included article, the following data items will be extracted based on the study Population Exposure Comparator Outcome: (1) Study information (author, year of publication, conflict of interest, ethical approval), (2) Study characteristics (study design and type, duration, data source, aim, recruitment, statistical analysis), (3) Population characteristics (description of main characteristics, treatment, comorbidities), (4) Exposure and outcomes (measurement of socioeconomic deprivation at individual, area or aggregate level, eg, IMD, Carstairs, Townsend Index), (5) Effect size (adjusted and unadjusted effect sizes) and uncertainty, (6) Main findings and (7) Key conclusions (online supplemental file 3).

### Risk of bias and quality assessment in individual studies

We evaluated tools available for the assessment of RoB of non-randomised studies: Newcastle-Ottawa Scale (NOS) and ROBINS-I.^52 53^ The NOS is a widely used tool for assessing the risk of bias of observational studies (both cohort and cross-sectional), the type of study design this review will focus on.^54^ Its advantages (eg, speed, accuracy, adaptability) and limitations (eg, subjectivity) are well-known. ROBIN-1 was excluded as it assumes a hypothetical intervention when socioeconomic deprivation is not an intervention which can be manipulated.

The risk of bias for the included studies will be assessed by two reviewers (ZD and SC) using the NOS which assesses the quality of non-randomised studies based on their selection, comparability and outcome/exposure characteristics. High-quality choices within each domain are awarded stars which are converted to ratings: good, fair and poor, which will be recorded on the data extraction form. The rating will not lead to any study being excluded, but caution will be used during data analysis for studies rated poor and fair quality.

We will report RoB of each study and explain the score. The RoB score will be incorporated into the narrative synthesis informing interpretation and discussion.

Any discrepancies in RoB and quality assessment will be resolved through discussion between the reviewers and the project leads (JSC and NA).

Methodological Index for Non-Randomised Studies will be used to assess the quality of the included studies.^55^

### Outcomes and prioritisation

Main outcomes:

1. SCT transplant referral (referral to a specialist tertiary centre/transplant centre), including SCT referral rates %, number of SCT referrals, likelihood of SCT referral OR, 95% CI, stratified according to socioeconomic deprivation.
2. SCT utilisation/rate, including transplant rate %, number of SCT, likelihood of SCT, stratified according to socioeconomic deprivation.
3. Survival, including OS/mortality, transplant survival rates, % survival.

Additional outcomes of interest include:

1. Delayed diagnosis measured by multiple GP visits/multiple symptoms (number or %), place of presentation (presents with symptoms at GP, through other investigations, symptoms require A&E admission, place of diagnosis, number of diagnosis in the following setting Accident & Emergency, GP, secondary care, advanced disease/genetic risk/grade of disease at presentation).
2. Treatments/interventions received (number or % patients receiving intensive chemotherapy/conditioning, systemic anti-cancer therapy).
3. Time between different stages of the treatment pathway.
4. Presence of comorbidities (Charlson Comorbidity Index, Haematopoietic Cell Transplantation-specific Comorbidity Index scoring systems used by Transplant Centres).

### Data synthesis

Narrative synthesis will be conducted on the findings from the review, where outcomes include delay in diagnosis/referral treatments/interventions received, referral to transplant, presence of comorbidities affecting transplant eligibility, transplant utilisation rates, waiting time to transplant, survival rates will be presented under broad headings with detailed summary of findings.

If data are available and homogeneous and there are at least three studies with comparable measurement of socioeconomic deprivation (individual/household, area) reporting the outcomes of interest, meta-analysis will be conducted. Only studies using the same study designs will be considered for pooling. Meta-analysis of Observational Studies in Epidemiology (MOOSE) recommendations will be used to guide the meta-analysis of observational studies.^56^

We will produce pooled ORs using a random or fixed-effect model to assess likelihood of transplant referral and/or utilisation, delayed diagnosis, comorbidity according to measures of socioeconomic deprivation (least deprived/most deprived).

Within each outcome, meta-analysis will follow for each individual/household, or area level of socioeconomic determinant exposure if there are three or more studies reporting sufficiently similar combinations of exposures and outcomes. Summaries of exposure effects for each study will be provided by calculating a pooled OR, HR, risk ratio (RR) and risk difference (RD). Tests will be applied for heterogeneity between included studies using the I² test statistic. Attention will be given to whether analyses adjust for key confounders, including ethnicity, age, sex, comorbidity and disease-related factors (eg, disease subtype or risk where reported). We will extract both unadjusted and the most fully adjusted estimates reported. In synthesis, priority will be given to analyses that adjust for key confounders, particularly ethnicity, given its known correlation with socioeconomic deprivation in the UK.

During synthesis, where studies do not adjust for ethnicity, findings from such studies will be interpreted cautiously as potentially confounded. Where sufficient data are available, we will explore results separately for studies that adjust for ethnicity versus those that do not.

In this review, we have broadly defined socioeconomic deprivation encompassing individual, household-level and area-level measures to include all data in this area. Both area-level and individual-level measures will be eligible for extraction and analysis. Where studies report categorical deprivation (eg, IMD quintiles) and continuous scores, both will be extracted and harmonised where possible. Multidimensional measures (eg, IMD domains) will be included in analysis only if an overall summary (IMD score) is reported.

We will group different deprivation measures according to area level (eg, IMD, Townsend, Carstairs) or individual levels (eg, income, education) and analyse outcomes according to these groups. As the different measures of area level deprivation (eg, IMD vs Townsend) measure different domains, reflecting different types of deprivation (Townsend/Carstairs: material vs IMD material/structural), we will further group findings according to a deprivation measure (eg, IMD or Townsend or education).

As the different deprivation measures are capturing different mechanisms, we will discuss the differences between the measures and how these differences may influence the findings.

Given the anticipated heterogeneity in study design, exposure definition, outcome/exposure measurement and adjustment strategies, results may not be suitable for pooling. Quantitative synthesis may be possible if there is homogeneity in the measure of socioeconomic deprivation conceptually and numerically across the same outcomes (eg, OR of SCT/emergency diagnoses reported by IMD quintile), same adjustments made, low RoB, complete effect estimates and same study design. If these criteria are not met, we will aim to conduct a quantitative narrative analysis, reporting effect sizes, direction of the effect (number of studies reporting delayed diagnoses or lower likelihood of receiving SCT in more deprived vs less deprived), relate findings to RoB and compared adjustments across studies.

In absence of heterogeneity in at least 10 studies, publication bias will be assessed using the Egger’s regression test.

### Confidence in cumulative evidence

The certainty of evidence for each outcome will be assessed using the Grades Recommendations, Assessment, Development and Evaluation (GRADE) approach.^57^ As most included studies are expected to be observational (registry-based or cohort), evidence will begin at a low certainty rating. Certainty may be downgraded for risk of bias (eg, incomplete adjustment for confounders), inconsistency (variation in effect estimates), indirectness (differences in definitions of outcomes), imprecision (wide CIs or small samples), or publication bias. Where appropriate, evidence may be upgraded if there is a large, consistent effect, a dose–response gradient, or if residual confounding would likely reduce the observed association. Certainty of evidence will be graded as high, moderate, low or very low for each primary and additional outcome (delay in diagnosis/referral, treatments/interventions received, referral to transplant, presence of comorbidities affecting transplant eligibility, access to transplant, transplant utilisation rates, waiting time to transplant and survival rates).

### Measurements of treatment effect

For binary data, pooled OR with 95% CIs or other effect estimates will be presented. For continuous data, we will use the mean difference with 95% CIs.

### Subgroup analysis

If data are available, analysis of inequities at different points in the SCT treatment pathway for haematological malignancies will be conducted.

## Ethics and dissemination

Ethical approval is not required for this study as it is a secondary analysis of published data.

We are not aware of any ethical concerns regarding stem cell transplant or treatment of donors in the studies included in this research. This systematic review will retrieve, screen and extract data from UK research. Stem cell transplantation and the treatment of donors in the UK follow the National Health Service (NHS) and NHS Blood and Transplant (NHSBT) guidelines and policies and are regulated by the Human Tissue Authority (HTA) and Medicines and Healthcare Products Regulatory Agency (MHRA). The data which will be used in this review will consist of data from NHS digital/NHS England or single NHS centre data, which will follow the guidelines and regulations above.

The findings will be submitted for peer-reviewed publication, shared at conferences and disseminated to both clinical and patient groups including National (Black, Asian, Mixed Race and Minority Ethnic) BAME Transplant Alliance (NBTA) and National Institute for Health Research Blood and Transplant Research Unit (BTRU) in Precision Transplant and Cellular Therapeutics.

### Patient and public involvement

Patients and the public participated in the development of the review. This was undertaken with the support of a patient and public involvement and engagement (PPIE) Steering Committee made up of six representatives from transplant charities (Team Margo, African Caribbean Leukaemia Trust, research groups and stem cell donor registry in the UK (NHSBT, Anthony Nolan). There was a mix of ethnic groups and experience represented among the members including a patient and two caregivers. Following the presentation of the rationale and preliminary ideas for this review to the group, the scope and objectives of the review were discussed. While the PPIE steering committee thought the objectives and scope were appropriate, they emphasised the need to explore the link between ethnicity and socioeconomic deprivation. Based on their feedback, we will ensure that this review also reports outcomes where the impact of socioeconomic deprivation on the blood cancer patient pathway is described within different ethnic minority groups if these data are available. The PPIE group will provide their lived experience to the interpretation and meaning of the results, whether the findings match their experience and make sense, highlight where the evidence does not match their experience and what these findings could mean in real life. We plan to discuss some of the mechanisms behind the quantitative findings. The group may help prioritise the findings into which matter the most to patients at different stages of the treatment pathway. Patients and members of the public will not be involved in the study selection or extraction phases but will be involved in the evidence synthesis stage of the review and this will be documented according to Patient Student Partner Activity log. The final review and lay summary will be supported by the PPIE Steering Committee and disseminated via publications in scientific journals, conference presentations and to the wider community at patient facing meetings such NBTA and the BTRU.

## Supplementary material

10.1136/bmjopen-2025-111282 online supplemental file 1

10.1136/bmjopen-2025-111282 online supplemental file 2

10.1136/bmjopen-2025-111282 online supplemental file 3
