# Supplementary material for: Role of socioeconomic deprivation on inequities in care outcomes of haematological malignancies treated with stem cell transplant in the UK: a systematic review protocol
Source: BMJ Open. 2026 Jul 10;16(7):e111282. doi: 10.1136/bmjopen-2025-111282 (PMC13358268; doi:10.1136/bmjopen-2025-111282)
Supplement: Supplementary file 4 [file bmjopen-2025-111282.reviewer_comments.pdf]

PEER REVIEW HISTORY

BMJ Open publishes all reviews undertaken for accepted manuscripts. Reviewers are asked to complete a checklist review form and are provided with free text boxes to elaborate on their assessment. These free text comments are reproduced below.

ARTICLE DETAILS

Title (Provisional)

The role of socioeconomic deprivation on inequities in care outcomes of haematological malignancies, treated with stem cell transplant in the UK: A Systematic Review Protocol

Authors

Deplano, Zareen; Cusworth, Samuel; Doree, Carolyn; Griffin, James; Adderley, Nicola; Chandan, Joht Singh

---

VERSION 1 - REVIEW

**Reviewer**	**1**
**Name**	**Roberts, Nia**
**Affiliation**	**University of Oxford, UK, Bodleian Health Care Libraries,**
**Date**	**15-Dec-2025**
**COI**	**None**

I have reviewed the search methods for the protocol and suggest a few minor changes:

- Please give detail of which platforms will be used for searching Medline & Embase. It looks like Ovid for Medline, but please make this explicit.

- As you intend to exclude RCTs, what is the value of searching CENTRAL? The same could be the same for Clinicaltrials.gov and WHO ICTRP.

- Please list which Proquest databases you intend to search.

- Please list which Web of Science databases you intend to search.

The search strategy for Medline is well constructed and there are no apparent errors. The UK filter developed by NICE seems to have been used to restrict to the UK, please give the reference to the development/validation paper. If any of the search blocks have been used for other reviews or adapted from other publiched filter, please put a reference to these.

**Reviewer**	**2**
**Name**	**Gomez-Almaguer, David**

**Affiliation**	**Univ Autonoma Nuevo Leon, hematologia**
**Date**	**26-Jan-2026**
**COI**	**None**

It is very difficult to obtain results if the auto vs allo HSCT are in the same protocol

Allogeneic HSCT is very different from autologous, Therefore the authors must consider including only one kind of transplant. Allo HSCT could be done using different sources of stem cells : Cord, identical related or unrelated and haploidentical. Autologous HSCT is easy and more uniform to be analyzed.

**Reviewer**	**3**
**Name**	**Raghav , Pawan K**
**Affiliation**	**University of California System, Surgery ITL**
**Date**	**26-Jan-2026**
**COI**	**None**

This protocol addresses an important and timely question regarding the role of socioeconomic deprivation in inequities across the stem cell transplant (SCT) pathway for hematological malignancies within the UK. The manuscript is highly relevant to NHS policy priorities, including Core20PLUS5 and equity of access to specialist cancer care. The protocol is generally well structured, registered prospectively, and adheres to PRISMA-P guidance. The comprehensive search strategy, inclusion of patient and public involvement, and focus on a universal healthcare system are notable strengths. However, several methodological and conceptual issues require clarification or refinement to ensure the review's feasibility, interpretability, and contribution beyond existing UK registry-based analyses. In particular, clearer operationalisation of socioeconomic deprivation, management of confounding (notably ethnicity and disease risk), justification for exclusions, and feasibility of meta-analysis are required.

Major comments

1. The concept of "socioeconomic deprivation" is very broadly defined, encompassing individual-, household-, and area-level measures, yet there is insufficient detail on how heterogeneity across these constructs will be handled analytically. Without clearer prioritisation or stratification, pooling or even narrative comparison across fundamentally different deprivation metrics (e.g., IMD vs income vs education) risks conceptual dilution. The authors should pre-specify a hierarchy or framework for deprivation measures (e.g., area-level vs individual-level) and clarify how discordant findings across levels will be interpreted.

2. Although the review aims to examine inequities “at all stages” of the SCT pathway, many proposed outcomes (e.g., delayed diagnosis, referral patterns, comorbidities) are upstream of transplant eligibility and may not be captured in SCT-focused datasets. The feasibility of identifying UK studies that simultaneously link deprivation, referral, eligibility assessment, and transplant receipt should be discussed more explicitly, including the likelihood that different outcomes will be informed by distinct and non-overlapping study populations.

3. The protocol states that qualitative and mixed-methods studies are excluded due to resource constraints. Given that mechanisms underlying socioeconomic inequities are a stated objective, excluding qualitative evidence substantially limits the ability to address this aim. The authors should either (a) justify more clearly why mechanisms can be adequately inferred from observational quantitative studies alone, or (b) reframe the second objective to reflect a more descriptive or hypothesis-generating intent.

4. The interaction between socioeconomic deprivation and ethnicity is acknowledged but insufficiently integrated into the analytic plan. Given the strong correlation between deprivation and ethnicity in the UK context, failure to address confounding and effect modification may lead to misleading conclusions. The protocol should specify whether studies adjusting for ethnicity will be prioritized, how unadjusted estimates will be treated, and whether subgroup or stratified analyses by ethnicity are planned a priori rather than opportunistically.

5. The decision to exclude post-transplant outcomes such as graft-versus-host disease and infection is not adequately justified. These outcomes may be influenced by socioeconomic factors (e.g., housing conditions, caregiver support, access to follow-up care) and are relevant to equity in transplant benefit, not merely access. The authors should clarify whether the review’s scope is intentionally limited to access and survival only, and justify why intermediate or morbidity-related outcomes are excluded.

6. The planned meta-analysis appears optimistic given the anticipated heterogeneity in study design, exposure definition, outcome measurement, and adjustment strategies. The protocol should more clearly state under what realistic circumstances quantitative synthesis would be abandoned in favour of narrative synthesis, and how selective reporting of pooled estimates will be avoided.

7. The use of the Newcastle–Ottawa Scale alone may be insufficient for assessing bias in complex observational studies of health inequities, particularly regarding residual confounding and selection bias. The authors should justify the choice of NOS over alternative or supplementary tools and clarify how risk-of-bias judgments will influence interpretation, especially if no studies are excluded based on quality.

8. The inclusion criteria allow for systematic reviews and research syntheses, yet the planned data synthesis does not explain how overlapping primary studies will be managed or whether such reviews will be used only for reference mining. This ambiguity should be resolved to avoid double counting of evidence.

Minor comments

9. The abstract would benefit from greater clarity on whether the review focuses primarily on access to SCT, utilisation, or outcomes after transplant, as the current wording suggests all three without clear prioritisation.

10. The rationale section could be strengthened by more clearly distinguishing how this review advances beyond existing UK registry analyses, rather than reiterating known inequities.

11. The exclusion of non-English language studies should be acknowledged as a potential source of bias in the limitations section, even if unavoidable.

12. The planned use of Google Scholar limited to the first 10 pages may miss relevant grey literature; a brief justification for this threshold would be helpful.

13. Some outcome definitions (e.g., “access to SCT” versus “utilisation”) overlap conceptually and should be more clearly delineated to avoid redundancy during extraction and synthesis.

14. The protocol would benefit from a short clarification on how overlapping cohorts (e.g., multiple publications from the same registry or time period) will be identified and managed.

15. The patient and public involvement section is strong, but it would be useful to specify how PPI input will concretely influence interpretation of findings rather than dissemination alone.

VERSION 1 - AUTHOR RESPONSE

Reviewer: 1<br>Ms. Nia Roberts, University of Oxford, UK<br>I have reviewed the search methods for the protocol and suggest a few minor changes:	
Please give detail of which platforms will be used for searching Medline & Embase. It looks like Ovid for Medline, but please make this explicit.	• MEDLINE (Ovid, 1946 onwards)<br>• Embase (Ovid, 1974 onwards). This is specified in supplementary file 2.<br>• Added to protocol
As you intend to exclude RCTs, what is the value of searching CENTRAL? The same could be the same for Clinicaltrials.gov and WHO ICTRP.	The decision to exclude RCTs, based on the rationale that the data in this area is likely be found in observational studies, was made after the search strategy was constructed and searches performed. We plan to exclude RCTs at the screening stage, documenting this in the subsequent results paper. The exclusion of RCTs have been removed from the protocol.
- Please list which Proquest databases you intend to search	Listed under the section ‘information sources’ and in supplementary file 2. Added to the protocol.
Please list which Web of Science databases you intend to search.	Listed under the section ‘information sources’ and in supplementary file 2

The search strategy for Medline is well constructed and there are no apparent errors. The UK filter developed by NICE seems to have been used to restrict to the UK, please give the reference to the development/validation paper. If any of the search blocks have been used for other reviews or adapted from other publiched filter, please put a reference to these.	CD: This is the list of 12 databases from the Web of Science:<br><br>Web of Science Core Collection<br><br>Biological Abstracts<br><br>BIOSIS Citation Index<br><br>Current Contents Connect<br><br>Data Citation Index<br><br>Grants Index]<br><br>KCI-Korean Journal Database<br><br>Policy Citation Index<br><br>Preprint Citation Index<br><br>ProQuest Dissertations & Theses Citation Index<br><br>Research Commons<br><br>SciELO Citation Index.<br>CD: The NICE UK geographic search filters for Ovid MEDLINE and Ovid Embase have been validated by the following papers:<br><br>1. Ayiku L, Levay P, Hudson T, Craven J, Barrett E, Finnegan A, Adams R. The MEDLINE UK filter: development and validation of a geographic search filter to retrieve research about the UK from OVID MEDLINE. Health Information and Libraries Journal. 2017, 34(3):200-216. doi: 10.1111/hir.12187. PMID: 28703418.<br><br>2. Ayiku L, Levay P, Hudson T, Craven J, Finnegan A, Adams R, Barrett E. The Embase UK filter: validation of a geographic search filter to retrieve research about the UK from OVID Embase. Health Information and Libraries Journal. 2019, 36(2):121-133. doi: 10.1111/hir.12252. PMID: 30912233.

<table>
<tr><td></td><td>3. Ayiku L, Levay P, Hudson T, Finnegan A. The NICE UK geographic search filters for MEDLINE and Embase (Ovid): Post-development study to further evaluate precision and number-needed-to-read when retrieving UK evidence. Research Synthesis Methods. 2020, 11(5):669-677. doi: 10.1002/jrsm.1431. PMID: 32618106.

All of the other search blocks were designed by the Information Specialist specifically for this review, so require no additional references.

Added to supplementary file 2.</td></tr>
<tr><td colspan="2">Reviewer: 2
Dr. David Gomez-Almaguer, Univ Autonoma Nuevo Leon</td></tr>
<tr><td>Comments to the Author:
It is very difficult to obtain results if the auto vs allo HSCT are in the same protocol Allogeneic HSCT is very different from autologous, Therefore the authors must consider including only one kind of transplant. Allo HSCT could be done using different sources of stem cells : Cord, identical related or unrelated and haploidentical. Autologous HSCT is easy and more uniform to be analyzed.</td><td>There is limited data in this setting and therefore we plan to describe the little there is but if we find more data, we will report allo-HSCT and auto-HSCT separately.</td></tr>
<tr><td colspan="2"></td></tr>
<tr><td>Comments to the Author:
This protocol addresses an important and timely question regarding the role of socioeconomic deprivation in inequities across the stem cell transplant (SCT) pathway for hematological malignancies within the UK. The manuscript is highly relevant to NHS policy priorities, including Core20PLUS5 and equity of access to specialist cancer care. The protocol is generally well structured, registered prospectively, and adheres to PRISMA-P guidance. The comprehensive search strategy, inclusion of patient and public involvement, and focus on a universal healthcare system are notable strengths. However, several methodological and conceptual issues require clarification or refinement to ensure the review’s feasibility, interpretability, and contribution beyond existing UK registry-based analyses. In particular, clearer operationalisation of socioeconomic deprivation, management of</td><td>We thank the reviewer for this important point. We have strengthened the protocol to clarify the operationalisation of socioeconomic deprivation, our handling of key confounders (including ethnicity and disease-related factors), the rationale for exclusions, and the realistic conditions under which meta-analysis will be undertaken.

Specifically:

1. Operationalisation of socioeconomic deprivation
Socioeconomic deprivation will be defined using validated measures and grouped a priori into:
(i) area-level composite indices (e.g., Index of Multiple Deprivation and national equivalents)</td></tr>
</table>

confounding (notably ethnicity and disease risk), justification for exclusions, and feasibility of meta-analysis are required.	(ii) area-level material deprivation indices (e.g., Townsend, Carstairs), and (iii) individual-level socioeconomic indicators (e.g., income, education, occupation). Added under 'Eligibility criteria'section.<br>Where studies report both categorical and continuous deprivation measures, we will extract both and harmonise within comparable groupings where feasible. Multidimensional measures (e.g., IMD domains) will be included only where an overall summary score is reported. Findings will be synthesised within these groupings rather than pooled across fundamentally different constructs. Added to 'Data synthesis' section<br><br>**2. Management of confounding (ethnicity and disease risk)**<br>We will extract both unadjusted and the most fully adjusted estimates reported. Attention will be given to whether analyses adjust for key confounders, including ethnicity, age, sex, comorbidity, and disease-related factors (e.g., disease subtype or risk where reported). In synthesis, we will prioritise interpretation of adjusted estimates and clearly label unadjusted findings as potentially confounded. Where sufficient data exist, we will explore results separately for studies that adjust for ethnicity versus those that do not. Added to 'Data synthesis' section<br><br>**3. Justification of exclusions**<br>Exclusions have been clarified in the Methods and are based either on (i) alignment with the review's primary focus on inequities across the SCT access and utilisation pathway, or (ii) feasibility and resource considerations typical of systematic review conduct.<br><br>**4. Feasibility of meta-analysis**<br>We agree that substantial heterogeneity

	is likely. Meta-analysis will therefore be undertaken only where ≥3 studies are sufficiently comparable in deprivation measure, outcome definition, and analytic approach. Where these conditions are not met, findings will be synthesised narratively using structured methods. This is described in the 'Data synthesis' section
Major comments	
1. The concept of "socioeconomic deprivation" is very broadly defined, encompassing individual-, household-, and area-level measures, yet there is insufficient detail on how heterogeneity across these constructs will be handled analytically. Without clearer prioritisation or stratification, pooling or even narrative comparison across fundamentally different deprivation metrics (e.g., IMD vs income vs education) risks conceptual dilution. The authors should pre-specify a hierarchy or framework for deprivation measures (e.g., area-level vs individual-level) and clarify how discordant findings across levels will be interpreted.	In this review, we have broadly defined socioeconomic deprivation encompassing individual, household- and area-level measures to include all data in this area.<br><br>We will group different deprivation measures according to area level (e.g. IMD, Townsend, Carstairs) or individual levels (e.g. income, education) and analyse outcomes according to these groups. As the different measures of area level deprivation (e.g. IMD vs Townsend) measure different domains, reflecting different type of deprivation (Townsend: material vs IMDL structural), we will further group findings according to a deprivation measure (e.g. IMD or Townsend or education).<br><br>As the different deprivation measures are capturing different mechanisms, we will discuss the differences between the measures and how these differences may influence the findings.<br><br>Added to 'Data analysis' section
2. Although the review aims to examine inequities "at all stages" of the SCT pathway, many proposed outcomes (e.g., delayed diagnosis, referral patterns, comorbidities) are upstream of transplant eligibility and may not be captured in SCT-focused datasets. The feasibility of identifying UK studies that simultaneously link deprivation, referral, eligibility assessment, and transplant receipt should be discussed more explicitly, including the likelihood that different outcomes will be informed by distinct and non-overlapping study populations.	The outcomes (e.g., delayed diagnosis, referral patterns, comorbidities) upstream of transplant eligibility may not be captured in SCT-focused datasets and are more likely to be informed by distinct and non-overlapping study populations. Our screening has been designed to include studies reporting on haematological malignancies and/or SCT study populations to ensure we include all available data<br><br>Added to protocol under 'Study Selection Process'
3. The protocol states that qualitative and mixed-methods studies are excluded due to resource constraints. Given that	We thank the reviewer for this important observation. We acknowledge that

mechanisms underlying socioeconomic inequities are a stated objective, excluding qualitative evidence substantially limits the ability to address this aim. The authors should either (a) justify more clearly why mechanisms can be adequately inferred from observational quantitative studies alone, or (b) reframe the second objective to reflect a more descriptive or hypothesis-generating intent.	exclusion of qualitative and mixed-methods studies limits the ability to make causal inferences about mechanisms underlying socioeconomic inequities.<br><br>This review is intentionally focused on quantitative observational evidence to characterise the distribution and magnitude of inequities across the SCT pathway within the UK. Added to the 'Eligibility criteria' section<br><br>In response to the reviewer's suggestion, we have reframed the second objective to clarify that the review will take a descriptive and hypothesis-generating approach to potential mechanisms, based on patterns observed in the quantitative literature and identified evidence gaps.
4. The interaction between socioeconomic deprivation and ethnicity is acknowledged but insufficiently integrated into the analytic plan. Given the strong correlation between deprivation and ethnicity in the UK context, failure to address confounding and effect modification may lead to misleading conclusions. The protocol should specify whether studies adjusting for ethnicity will be prioritized, how unadjusted estimates will be treated, and whether subgroup or stratified analyses by ethnicity are planned a priori rather than opportunistically.	We thank the reviewer for highlighting this important issue. We agree that socioeconomic deprivation and ethnicity are closely correlated in the UK context and that failure to account for this may bias interpretation.<br><br>In the revised protocol, we have strengthened the analytic plan. We will extract both unadjusted and the most fully adjusted estimates reported and will prioritise interpretation of analyses that adjust for ethnicity and other key confounders (e.g., age, sex, comorbidity, and disease-related factors where available).<br><br>During synthesis noted where studies do not adjust for ethnicity, and findings from such studies will be interpreted cautiously as potentially confounded. Where sufficient data are available, we will explore results separately for studies that adjust for ethnicity versus those that do not. These changes have been incorporated into the 'Methods' and 'Data synthesis' section
5. The decision to exclude post-transplant outcomes such as graft-versus-host	This review is focussed on inequities in access to stem cell transplant from the

disease and infection is not adequately justified. These outcomes may be influenced by socioeconomic factors (e.g., housing conditions, caregiver support, access to follow-up care) and are relevant to equity in transplant benefit, not merely access. The authors should clarify whether the review's scope is intentionally limited to access and survival only and justify why intermediate or morbidity-related outcomes are excluded.	point of diagnosis to transplant, hence post-transplant outcomes were intentionally excluded. Lack of access to treatment impacts on the survival of patients, especially where the treatment is a potential cure such as in adverse risk AML, high risk MDS, myeloma. There is mixed results on the role of socioeconomic deprivation on access and survival in the haematological malignancies setting in the UK, hence we plan to synthesise the data in this space. This is aligned with reports published in this setting as detailed in the protocol (e.g. The UK stem cell strategic forum report, 2022, APPG 2021 and NHS Long term plan)<br><br>We agree that socioeconomic deprivation influences post-transplant outcomes resulting in inequities, but we are unable to expand the scope due to resource constraints<br><br>Added to protocol under 'Eligibility criteria'
6. The planned meta-analysis appears optimistic given the anticipated heterogeneity in study design, exposure definition, outcome measurement, and adjustment strategies. The protocol should more clearly state under what realistic circumstances quantitative synthesis would be abandoned in favour of narrative synthesis, and how selective reporting of pooled estimates will be avoided.	Given the anticipated heterogeneity in study design, exposure definition, outcome/exposure measurement, and adjustment strategies, results may not be suitable for pooling. Quantitative synthesis may be possible if there is homogeneity in the measure of socioeconomic deprivation conceptually and numerically across the same outcomes (e.g. OR of SCT/emergency diagnoses reported by IMD quintile), same adjustments made, low RoB, complete effect estimates and same study design. If these criteria are not met, we will aim to conduct a quantitative narrative analysis, reporting effect sizes, direction of the effect (number of studies reporting delayed diagnoses or lower likelihood of receiving SCT in more deprived vs less deprived), relate findings to RoB, compared adjustments across studies.<br><br>Added in 'Data synthesis' section
7. The use of the Newcastle–Ottawa Scale alone may be insufficient for assessing bias in complex observational studies of health inequities, particularly regarding residual confounding and selection bias. The authors should justify the choice of NOS over alternative or supplementary tools and clarify how risk-of-bias judgments will	We evaluated tools available for the assessment of RoB of non-randomised studies: NOS and ROBINS-I. The NOS is a widely used tool for assessing the risk of bias of observational studies (both cohort and cross-sectional), the type of study design this review will focus on. Its advantages (e.g. speed, accuracy,

influence interpretation, especially if no studies are excluded based on quality.	adaptability) and limitations (e.g. subjectivity) are well-known. ROBIN-1 was excluded as it assumes a hypothetical intervention when socioeconomic deprivation is not an intervention which can be manipulated. We chose NOS as it does not require resource intensive and methodological expertise.<br><br>We will report RoB of each study and explain the score. The RoB score will be incorporated into the narrative synthesis informing interpretation and discussion. RoB will be supported using MINORS to assess quality of the included studies
8. The inclusion criteria allow for systematic reviews and research syntheses, yet the planned data synthesis does not explain how overlapping primary studies will be managed or whether such reviews will be used only for reference mining. This ambiguity should be resolved to avoid double counting of evidence.	Systematic reviews and research syntheses will be included and used to identify primary studies, as a source of references. They may also be used in the introduction and/or discussion of the review.<br><br>Added under 'Eligibility criteria' section
Minor comments	
9. The abstract would benefit from greater clarity on whether the review focuses primarily on access to SCT, utilisation, or outcomes after transplant, as the current wording suggests all three without clear prioritisation.	We have added 'from the point of diagnosis to transplant' to the aim at the end of the first paragraph in the introduction of the abstract. We have also removed the outcome 'access to transplant' and added 'post-transplant outcomes are beyond the scope of this review' to the methods section of the abstract
10. The rationale section could be strengthened by more clearly distinguishing how this review advances beyond existing UK registry analyses, rather than reiterating known inequities.not well understood,	Rationale section revised
11. The exclusion of non-English language studies should be acknowledged as a potential source of bias in the limitations section, even if unavoidable.	Due to resource constraints, non-English language studies will be excluded.<br><br>Added to the limitations section
12. The planned use of Google Scholar limited to the first 10 pages may miss relevant grey literature; a brief justification for this threshold would be helpful.	Methodology papers (Briscoe, 2017; Haddaway et al, 2015) recommend screening the first 200-300 results. Our lower threshold (100 results) was selected based on our available resources and the use of other grey literature source, namely ProQuest Dissertations & Theses Citation Index.<br><br>Added under 'Information Sources' section

13. Some outcome definitions (e.g., "access to SCT" versus "utilisation") overlap conceptually and should be more clearly delineated to avoid redundancy during extraction and synthesis.	We agree that 'access to SCT' and 'SCT utilisation' overlap. We use access to SCT covers both SCT referral and rates/utilisation and therefore we have removed this as an outcome. Instead, we will discuss access to SCT in the 'results' and 'discussion' section, using results on SCT referrals and utilisation to discuss 'access to SCT'
14. The protocol would benefit from a short clarification on how overlapping cohorts (e.g., multiple publications from the same registry or time period) will be identified and managed.	Following Cochrane handbook guidance, we will compare multiple publications from the same study based on criteria including the authors names, location/setting, number of participants, baseline data, number of participant and date and duration of the study. If the publications are identified to be from the same study, they will be merged into one study record, data will be extracted from both, We will note any inconsistencies and the authors will be contacted.<br><br>Added to 'Study selection process' section
15. The patient and public involvement section is strong, but it would be useful to specify how PPI input will concretely influence interpretation of findings rather than dissemination alone.	The PPIE group will provide their lived experience to the interpretation and meaning of the results, whether the findings match their experience and make sense, highlight where the evidence does not match their experience and what these findings could mean in real life. We plan to discuss some of the mechanisms behind the quantitative findings. The group may help prioritise the findings into which matter the most to patients at different stages of the treatment pathway.<br><br>Added to 'Patient and Public involvement' section

VERSION 2 - REVIEW

Reviewer **1**

Name **Roberts, Nia**

Affiliation **University of Oxford, UK, Bodleian Health Care Libraries,**

Date **08-Apr-2026**

COI

Comments on the main text:

The range of databases included is impressive, however, the reporting of which databases is available via which platform is a little confusing in the main text under Information Sources (p15), particularly for Proquest and Web of Scienc.

There are 2 options to provide clarity.

(1)If word count is not an issue, I would encourage you to improve consistency in use of () in distinguishing database from platform and the introduction of added punctuation to illustrate which databases are being searched via Proquest and Web of Science. Clarity could also be improved if unnecessary database were removed from the Web of Science search e.g. grants index, Korean database are unlikely to provide relevant data.

(2) Condence the reporting of the databases searched via Proquest and Web of Science and refer readers to the Supplementary Material for additional information on databases included. There's already a much more clearly laid out list of databases in this section, but it would need to be expanded to include all the Web of Science databases.

Please give the full database names for CENTRAL and CDSR and indicate that it's being searched via Cochrane Library, Wiley.

Make it clear that ClinicalTrials.gov and WHO ICTRP are being searched separately, currently looks like it's part of the Web of Science database search.

Comments on the Supplementary Material:

The search strategies are detailed and comprehensive. I have a concern about one of the limits applied to the Embase search:

limit 59 to (english language and "remove medline records")

This limit risks missing relevant records that although existed in Medline were not captured by the Medline search, which were however captured by the subsequent Embase search due to the additional indexing. As Covidence deals well with duplicates, I would encourage you to remove this limit and allow Covidence to deal with de-duplication on import, rather than exclude on the search.

**Reviewer**	**2**
**Name**	**Gomez-Almaguer, David**
**Affiliation**	**Univ Autonoma Nuevo Leon, hematologia**
**Date**	**08-Apr-2026**
**COI**	

This manuscript presents a protocol concerning a systematic review regarding the role of socioeconomic deprivation in the inequities in access to SCT and its outcomes in the UK population with hematological malignancies. The paper approaches a relevant topic and is appropriate to admit.

The previous comments to the author have already been corrected, added to the protocol, or clarified according to the limitations in the responses from the applicants, and thus, it is adequate to accept.

VERSION 2 - AUTHOR RESPONSE

<table>
<tr><td colspan="2">Reviewer: 1
Ms. Nia Roberts, University of Oxford, UK
The range of databases included is impressive, however, the reporting of which databases is available via which platform is a little confusing in the main text under Information Sources (p15), particularly for Proquest and Web of Science.</td></tr>
<tr><td>Comments to the Author:
Comments on the main text:

The range of databases included is impressive, however, the reporting of which databases is available via which platform is a little confusing in the main text under Information Sources (p15), particularly for Proquest and Web of Science.

There are 2 options to provide clarity.

(1)If word count is not an issue, I would encourage you to improve consistency in use of () in distinguishing database from platform and the introduction of added punctuation to illustrate which databases are being searched via Proquest and Web of Science. Clarity could also be improved if unnecessary database were removed from the Web of Science search e.g. grants index, Korean database are unlikely to provide relevant data.

(2) Condence the reporting of the databases searched via Proquest and Web of Science and refer readers to the Supplementary Material for additional information on databases included. There's already a much more clearly laid out list of databases in this section,</td><td>Thank you for your feedback, we have amended using options 1 and 2.

Amended, page 13, under ’Information Sources’

Amended, page 13, under ’Information Sources’: (Supplementary File 2) added</td></tr>
</table>

but it would need to be expanded to include all the Web of Science databases.	
Please give the full database names for CENTRAL and CDSR and indicate that it's being searched via Cochrane Library, Wiley.	Amended under 'Information Sources', page 13
Make it clear that ClinicalTrials.gov and WHO ICTRP are being searched separately, currently looks like it's part of the Web of Science database search.	Amended under 'Information Sources', page 13
Comments on the Supplementary Material:<br><br>The search strategies are detailed and comprehensive. I have a concern about one of the limits applied to the Embase search:<br><br>limit 59 to (english language and "remove medline records")<br><br>This limit risks missing relevant records that although existed in Medline were not captured by the Medline search, which were however captured by the subsequent Embase search due to the additional indexing. As Covidence deals well with duplicates, I would encourage you to remove this limit and allow Covidence to deal with de-duplication on import, rather than exclude on the search.	Thank you for your suggestion. However, these search strategies have been very carefully designed and tested, for a topic which will inevitably produce a large number of results to screen, and the addition of this filter to the Embase strategy is there in order to prevent an additional 3,000+ irrelevant results being retrieved by the Embase search - many of which will not be removed by the Covidence de-duplicator, as they will not have been retrieved by searches in the other databases. We would therefore like to retain this limit to the Embase search please – the reasoning being that Embase has a far more liberal indexing system, which means that irrelevant results are retrieved far more readily in searches of this database, while our MEDLINE search strategy has been very carefully designed to target the more accurate and targeted MeSH indexing system within MEDLINE. By not using this filter in Embase, the advantage created by using a carefully constructed search strategy in MEDLINE will unfortunately be lost, so we would request please that we keep this limit on the search.<br><br>This has been added as a limitation of the review on page 4
Reviewer: 2<br>Dr. David Gomez-Almaguer, Univ Autonoma Nuevo Leon	
Comments to the Author:<br>This manuscript presents a protocol concerning a systematic review regarding the role of socioeconomic	We thank the reviewer for their comments which contributed towards an improvement of the manuscript

deprivation in the inequities in access to SCT and its outcomes in the UK population with hematological malignancies. The paper approaches a relevant topic and is appropriate to admit.<br><br>The previous comments to the author have already been corrected, added to the protocol, or clarified according to the limitations in the responses from the applicants, and thus, it is adequate to accept.	
